# Common variants of fetal and maternal complement genes in preeclampsia: pregnancy specific complotype

**DOI:** 10.1038/s41598-020-60539-9

**Published:** 2020-03-16

**Authors:** Manu Banadakoppa, Meena Balakrishnan, Chandra Yallampalli

**Affiliations:** Department of Obstetrics & Gynecology, Baylor College of Medicine, Houston, Texas USA

**Keywords:** Reproductive biology, Reproductive disorders

## Abstract

Preeclampsia (PE) is a pregnancy specific hypertensive disorder. If untreated PE leads to life threatening condition, eclampsia. Systemic complement activation levels are increased during pregnancy compared to non-pregnant women of childbearing age. In PE, systemic complement levels are further increased, and higher complement deposition has been observed on placentas. We hypothesize that combinations of common SNPs in maternal and fetal complement genes constitute pregnancy specific complotypes and predispose women to PE. In this study, we sequenced two maternal (factor H and C3) and one fetal (CD46) complement genes and identified a total of 9 common SNPs. Minor allele frequencies of two fetal CD46 SNPs were significantly higher in PE. Further, complotypes consisting of fetal CD46 variants and maternal CFH/C3 variants were highly prevalent in PE patients compared to normotensive pregnancies. Placental complement deposition and maternal alternative pathway 50 (AP50) values were higher in PE pregnancies. Irrespective of disease status, two CD46 variants were associated with reduced placental CD46 expression and one CFH variant was associated with increased maternal AP50 values.

## Introduction

Preeclampsia (PE) is a pregnancy specific, multifactorial and multisystem complication usually manifested late in pregnancy and occurs in 3–8% of all pregnancies. The clinical hallmark of PE is new onset of elevation in blood pressure sometimes accompanied by proteinuria, end-organ dysfunction and fetal growth restriction. If left untreated PE may lead to eclampsia, a serious condition that causes life threating seizures. Overall, 10–15% of maternal mortality is associated with PE and eclampsia^1^. In addition, PE accounts for a large portion of premature births since delivery of placenta and child is the only definitive cure. The exact etiology of PE is unknown, and its clinical course is extremely difficult to predict. However, PE shows a genetic inheritance pattern with penetrance of less than 50% and contributions from both maternal and fetal genes^2^. Several putative mechanisms have been implicated in the pathogenesis of PE including partly defined role of complement cascade activation. Excess placental production of soluble fms-like tyrosine kinase (sFLT1), an antagonist of vascular endothelial growth factor (VEGF) and placental growth factor (PlGF) has been shown to be associated with decreased circulating levels of VEGF and PlGF in preeclampsia patients^3^. Administration of sFLT1 to pregnant rats caused preeclampsia symptoms such as hypertension, proteinuria and glomerular endotheliosis^3^. Administration of sFLT1 in pregnant mice increased angiotensin II sensitivity and hypertension by impairing endothelial nitric oxide synthase (eNOS) system and causing oxidative stress in the vasculature^4^. The ratio of sFLT1 to PlGF has been shown to predict development of preeclampsia^5–7^. Complement cascade is an important arm of innate immune system that provides frontline defense against invading microbes and also involved in the clearance of damaged cells and cellular debris^8^. Complement is a cascade of proteolytic cleavages consisting of more than 30 effector and regulator proteins. Complement can be activated through classical (CP), lectin (LP) and alternative (AP) pathways. Upon activation soluble effector proteins in the serum bind to cell surface in a step wise manner resulting in the assembly of cellulolytic pore like structure membrane attack complex (MAC). The regulators as either circulating proteins in the serum or cell surface bound allow controlled complement propagation and play a role in the discrimination of healthy self-cells and damaged self or foreign cells. The CP and LP are initiated with the formation of multi protein complex classical pathway C3 convertase (C4bC2a). The AP is always active at a low rate due to tick-over mechanism and involves formation of alternative pathway C3 convertase (C3bBb). Following the cleavage of C3 by respective convertases all three pathways converge and proceed to terminal activation to form MAC. Complement activation in the fluid phase is regulated by factor H assisted proteolytic clearance by factor I. On cell surfaces, in addition to recruitment of these soluble proteins, surface bound molecules decay accelerating factor (DAF or CD55), membrane cofactor protein (MCP or CD46) and CD59 regulate complement activation^9^.

In normotensive pregnant women systemic complement activation is increased compared to non-pregnant women of child-bearing age^10^. Further, increase in systemic and placental complement activation has been observed in women who developed PE^11–14^. Single nucleotide variations (SNP) in the genes of maternal circulating complement regulators such as factor I and factor H have been shown to be prevalent in preeclampsia patients^15,16^. However, association studies of SNPs in maternal cell surface complement regulator CD46 yielded ambiguous results. In PROMISSE study A304V mutation in CD46 was found to be associated with PE^16^ whereas no such association was found in a Finnish cohort^17^. Recently, several synonymous and intronic common SNPs in maternal C3 gene have been shown to be associated with PE^18^. In silico analysis of these synonymous and intronic variations indicated a possible role in mRNA splicing or folding suggesting that those SNPS may have functional relevance.

Common variations in complement genes can act synergistically to increase the complement activity several fold compared to single variations. These combinations of variations defined as “complotypes” are thought to determine the complement activity in an individual and influence susceptibility to complement associated diseases^19^. Further, a novel complotype combination consisting of SNPs in factor B and factor H was found to be strongly associated with age related macular degeneration than individual SNPs^20^. In a recent study, we have found that complement activation induced secretion of sFLT1 from human primary syncytiotrophoblast cells in a dose dependent manner^21^ suggesting that the level of complement activation plays an important role in PE pathology. Based on our previous data and those from others we hypothesize that the level of complement activation in PE is determined by oligogenic inheritance where SNPs in fetal cell surface regulators combine with SNPs in maternal circulating regulators/effectors to constitute pregnancy specific risk complotypes. In this study we screened a retrospective cohort of PE pregnancies to identify SNPs in one fetal and two maternal complement genes and compared their frequencies with those from normotensive pregnancies. Since C3 is central effector molecule in the cascade, and  CD46 and factor H regulate C3 activation, we selected CD46 (fetal) and C3, factor H (maternal) for sequencing in this study.

## Results

### Identification of common SNPs in maternal-fetal dyad of complement genes

We initially Sanger sequenced three complement genes from 16 PE pregnancies. We identified a total of 9 SNPs in three genes after excluding the SNPs with minor allele frequency of less than 5%. The occurrence of these SNPs in 24 normotensive pregnancies were then examined. The demographic information of subjects in the study groups are given in Table 1. Among the 9 SNPs identified, minor allele frequencies of rs1962149 and rs7144 (fetal CD46 gene) were significantly higher (*p* = 0.002 and *p* = 0.009 respectively) in PE pregnancies compared to normotensive pregnancies (Table 2).

**Table 1:** Patient characteristics.

	Normotensive	Preeclampsia	*p*
Gestational age at delivery (weeks)	37.6 ± 0.47	36.9 ± 0.77	0.43
Maternal age (years)	27.04 ± 1.08	26.56 ± 1.27	0.93
BMI (kg/m^2^)	25.13 ± 1.63	28.3 ± 1.75	0.047
SBP (mm Hg)	118.75 ± 2.34	143.13 ± 4.81	<0.0001
DBP (mm Hg)	71.66 ± 1.66	83.66 ± 3.06	0.001
Race (n) White Hispanic (%) African American (%)	18 (75) 6 (25)	12 (75) 4 (25)	>0.99 >0.99

Values are mean ± SEM. Fisher’s exact test was performed to assess statistical significance.

**Table 2:** Minor allele frequencies of SNPs in preeclampsia and normotensive pregnancies.

Gene	SNP	Nucleotide change	Minor allele frequency		p*
			Preeclampsia	Control	
C3	rs2547438	T>C	0.166	0.217	0.769
	rs2230204	C>G	0.3	0.285	>0.9
	rs2230205	C>T	0.2	0.199	0.508
	rs344555	T>C	0.312	0.3	>0.9
CFH	rs800292	G>A	0.538	0.304	0.077
	rs1061147	A>C	0.656	0.76	0.325
	rs2274700	G>A	0.375	0.32	0.639
CD46	rs1962149	G>A	0.843	0.5	0.002
	rs7144	T>C	0.892	0.588	0.009

^*^Fisher’s exact test.

### Pregnancy specific risk complotypes

In order to examine if combinations of fetal and maternal SNPs constitute risk complotypes we analyzed genotype frequencies of identified SNPs. Since combinations of genotypes into complotypes composed of 3 or more SNPs required a large number of specimens to represent possible combinations for meaningful interpretation, we restricted our analysis to combinations consisting of 2 SNPs. Frequencies of combinations consisting of homozygous genotype (AA) of fetal CD46 rs1962149 and heterozygous/homozygous genotypes (GA/AA, AC/CC, GG/GA) of maternal factor H SNPs (rs800292, rs1061147, 2274700 respectively) were significantly higher in PE pregnancies compared to normotensive pregnancies (Table 3). Similarly, combinations containing homozygous genotype (CC) of fetal CD46 rs7144 and heterozygous/homozygous genotypes of maternal factor H SNPs were significantly more abundant in PE pregnancies compared to normotensive pregnancies (Table 3). Among fetal CD46-maternal C3 pairs, combination containing homozygous genotype (AA) of CD46 rs1962149 and heterozygous/homozygous genotypes (AG/GG) of C3 rs344555 showed significantly higher frequency in PE pregnancies (Table 4). Two of the combinations from CD46 rs7144, genotype (CC) with genotypes (GA/AA) of C3 rs2230205 and genotypes (AG/GG) of C3 rs344555 showed significantly higher frequency in PE pregnancies (Table 5). Together, these data suggested that complotypes consisting of homozygous genotypes of fetal CD46 SNPs and heterozygous/homozygous genotypes of maternal factor H or C3 predisposed women to develop preeclampsia.

**Table 3:** Genotype combinations of SNPs in fetal CD46 and maternal factor H.

CD46	CFH	NT (%)	PE (%)	p*
**rs1962149**	**rs800292**			
GA	GA/AA	6 (25)	1 (6)	0.209
AA	GA/AA	2 (8)	9 (56)	0.002
**rs1061147**				
GA	AC/CC	11 (46)	2 (13)	0.004
AA	AC/CC	4 (17)	10 (63)	0.006
**rs2274700**				
GA	GG/GA	6 (25)	2 (13)	0.44
AA	GG/GA	2 (8)	12 (75)	>0.0001
**rs7144**	**rs800292**			
TC	GA/AA	3 (13)	0 (0)	0.26
CC	GA/AA	3 (13)	9 (56)	0.005
**rs1061147**				
TC	AC/CC	6 (25)	0 (0)	0.064
CC	AC/CC	5 (21)	10 (63)	0.018
**rs2274700**				
TC	GG/GA	0 (0)	1 (6)	
CC	GG/GA	1 (4)	8 (50)	0.001

*Fisher’s exact test. NT-normotensive. PE-preeclampsia.

**Table 4:** Genotype combinations of fetal CD46 rs1962149 and SNPs in maternal C3.

CD46	C3	NT (%)	PE (%)	p*
**rs1962149**	**rs2547538**			
GA	AC/CC	4 (17)	0 (0)	0.14
AA	AC/CC	2 (8)	4 (25)	0.19
**rs2230204**				
GA	GA/AA	1 (4)	1 (6)	
AA	GA/AA	2 (8)	4 (25)	0.19
**rs2230205**				
GA	GA/AA	5 (21)	1 (6)	0.37
AA	GA/AA	0 (0)	3 (13)	0.056
**rs344555**				
GA	AG/GG	14 (58)	3 (13)	0.02
AA	AG/GG	4 (17)	11 (46)	0.002

*Fisher’s exact test. NT-normotensive. PE-preeclampsia.

**Table 5:** Genotype combinations of fetal CD46 rs7144 and SNPs in maternal C3.

CD46	C3	NT (%)	PE (%)	p*
**rs7144**	**rs2547538**			
TC	AC/CC	3 (13)	0 (0)	0.26
CC	AC/CC	2 (8)	4 (25)	0.19
**rs2230204**				
TC	GA/AA	1 (4)	0 (0)	
CC	GA/AA	1 (4)	4 (25)	0.13
**rs2230205**				
TC	GA/AA	3 (13)	0 (0)	0.26
CC	GA/AA	0 (0)	4 (25)	0.019
**rs344555**				
TC	AG/GG	7 (29)	1 (6)	0.2
CC	AG/GG	4 (17)	12 (75)	0.001

*Fisher’s exact test. NT-normotensive. PE-preeclampsia.

### Complement deposition on placentas

**Figure 1:**
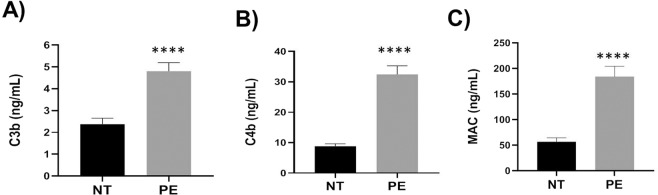
Complement depositions on placentas from preeclampsia (PE) patients were significantly higher compared to those from normotensive (NT) pregnancies. Complement levels in placental homogenates were measured by ELISA. (**A**) C3b, (**B**) C4b and (**C**) MAC. Values are presented as ng/mL ± SEM. n = 16 in preeclampsia and n = 24 in normotensive groups. ****p < 0.0001.

To assess if complement deposition is higher on placentas in PE study group compared to normotensive group we measured C3b, C4b and MAC levels in placental homogenates. Quantities of all three complement activation products measured were significantly higher (*p* < 0.0001) in placentas from PE group compared to those from normotensive group (Fig. 1). These data suggested that both classical and alternative pathways were activated at the fetal-maternal interface in PE pregnancies.

### Placental CD46 expression levels

**Figure 2:**
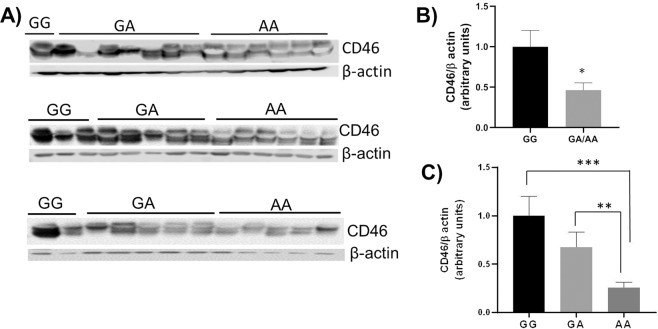
Placenta CD46 expression in pregnancies with or without rs1962149 (A > G). (**A**) Western blots showing placental CD46 levels (**B**) Density analysis of Western blots showing significantly higher levels of CD46 on placentas from GG compared to GA/AA genotypes. *p = 0.01. (**C**) The CD46 levels showed about 50% decrease with GA and 25% decrease with AA compared to GG genotype. n = 16–24, **p = 0.04, ***p = 0.007.

The levels of complement deposition on placentas in PE pregnancies were significantly higher compared to those from normotensive pregnancies. The allele frequencies of two fetal CD46 SNPs were also significantly higher in PE pregnancies. Further, homozygous genotypes of CD46 SNPs formed risk complotypes with maternal SNPs. However, both the CD46 SNPs were intronic or synonymous and therefore, unlikely affect CD46 protein structure and their cofactor activity. Therefore, we examined placental CD46 levels to assess if these two SNPs affected placental CD46 expression irrespective of PE pathology. Western blot results (Fig. 2A) indicated that the levels of CD46 on placentas from PE and normotensive pregnancies with hetero/homozygous genotype (GA/AA) of rs1962149 were significantly reduced (*p* = 0.01) compared to those from homozygous GG genotype (Fig. 2B). In addition, the minor allele (A) showed a dosage effect on CD46 levels (Fig. 2C). The placental CD46 levels were 50% (*p* = 0.04) and 25% (*p* = 0.007) reduced when the genotypes were heterozygous (GA) and homozygous (AA) respectively compared to GG genotype. Further analysis of Western blot results indicated that the rs7144 also affected expression levels of placental CD46 in a genotype dependent manner similar to that of rs1962149 (Figure not shown). These data suggested that the two CD46 SNPs present in PE and normotensive groups were associated with reduced placental expression of CD46.

### Maternal serum complement activation potential

**Figure 3:**
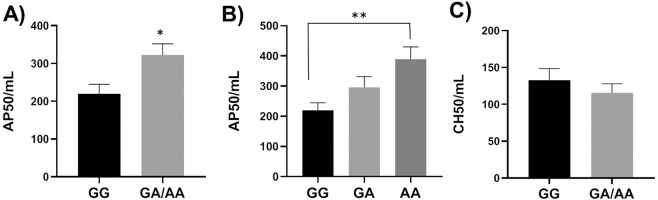
Alternative pathway 50 (AP50) and classical pathway 50 (CH50) values of maternal serum. (**A**) AP50 values of hetero/homozygous genotypes (GA/AA) of rs800292 were significantly higher compared to GG genotype. N = 13–17 in each group (**B**) Allele A of rs800292 showed dosage effect on AP50 values. N = 13 (GG),12 (GA), 5 (AA). (**C**) Genotypes of rs800292 showed no effect on CH50 values. *p = 0.03, **p = 0.02.

**Figure 4:**
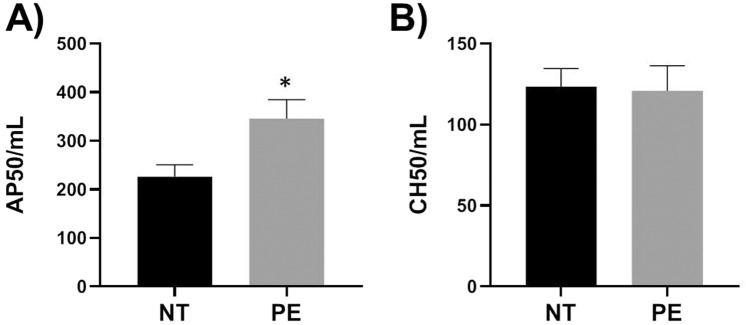
Alternative pathway 50 (AP50) and classical pathway 50 (CH50) values of serum from preeclampsia (PE) and normotensive (NT) pregnant women. N = 16–24 in each group. (**A**) AP 50 is significantly higher in PE group compared to NT. *p* = 0.017. (**B**) CH 50 was not different between two groups.

Since missense mutation rs800292 in maternal factor H constituted risk complotypes with fetal CD46 SNPs, we examined the alternative pathway 50 (AP50) and classical pathway 50 (CH50) values of maternal serum to assess if the SNP was associated with maternal complement activation potential irrespective of PE pathology. The AP50 values of maternal serum from hetero/homozygous genotypes (GA/AA) of rs8002922 was significantly higher (*p* = 0.03) compared to GG genotype (Fig. 3A) indicating that the SNP was associated with increased alternative pathway activation potential. In addition, the allele A showed a trend of dosage effect on AP50 values (Fig. 3B). However, the CH50 values of maternal serum were not different between the genotypes (Fig. 3C) indicating that rs800292 was not associated with classical pathway potential of maternal serum. Since minor allele frequency of rs800292 was higher in PE group (Table 2) we further analyzed AP50 and CH50 data to assess if complement activation potential of maternal serum was associated with disease status. The AP50 values of serum from mothers in PE group were significantly higher (*p* = 0.017) compared to those from normotensive group (Fig. 4A) suggesting that women who developed PE had higher alternative pathway complement activation potential. The CH50 values of maternal serum were not different between PE and normotensive groups (Fig. 4B) suggesting that classical pathway activation potential was not different between the two groups. Overall, these results suggested that missense mutation rs800292 of maternal factor H was associated with increased maternal alternative pathway activation potential and therefore, in combination with CD46 variants, rs800292 may contributed to the increased complement activation in PE patients.

## Discussion

We identified a total of 9 common SNPs of two maternal (factor H and C3) and one matched fetal (CD46) genes present in 16 PE pregnancies. Subsequently we examined if these SNPs were also present in 24 normotensive pregnancies. Allele frequencies of two CD46 SNPs, rs1962149 and rs7144 were significantly higher in PE pregnancies compared to normotensive pregnancies. Among these two SNPs, rs962149 was present in 76% of normotensive and 94% of PE pregnancies whereas rs7144 was present in 82% normotensive and 94% of PE pregnancies. Further analysis of genotype frequencies revealed that combinations of homozygous fetal CD46 SNPs and hetero/homozygous maternal factor H/C3 SNPs constituting complotypes were significantly higher in PE pregnancies. Together, these data suggested that complotypes consisting of fetal and maternal SNPs in complement genes may predispose women to develop PE. Our findings are similar to a recent report^20^ where complotype consisting of common SNPs in complement genes were shown to be very strongly associated with age related macular degeneration (AMD), a disease of eyes. Interestingly, heterozygous genotype (GA) of rs800292 (factor H) in AMD group showed strong association with complement activation levels compared to other genotypes of the AMD complotype. In the present study we also observed association of complotype containing GA genotype of rs800292 with PE indicating an important role of this SNP in placental complement activation.

All SNPs identified in the present study have been previously shown to be associated with various diseases involving complement cascade activation. Both the SNPs in fetal CD46 gene, rs1962149 and rs7144 were present in intronic regions and therefore, may not directly affect its cofactor activity in the clearance of deposited complement products. However, these two SNPs were part of MCPggaac haplotype that has been reported to be associated with atypical hemolytic uremic syndrome^22,23^. The MCPggaac haplotype present in the regulator of complement activation (RCA) locus of chromosome 1q32 includes SNPs in several complement genes that show very strong linkage disequilibrium (LD). Minor alleles of two additional common SNPs of the MCPggaac haplotype, rs2796267 and rs2796268 present in the promoter region of CD46 gene reduce expression of CD46 by 25%^22^. Consistent with previous report, we observed a dosage effect of allele A (rs1962149) on placental CD46 expression with 50% and 25% reduction when the genotypes were GA and AA respectively compared to CD46 levels in GG genotype. We assume that CD46 SNPs identified in our cohort may not be causal in nature but their association with PE may be due to strong LD with causal SNPs in MACPggaac haplotype. The reduced CD46 levels due to allele A (rs1962149) was not unique to placentas from PE pregnancies. The placentas from normotensive pregnancies also showed reduced levels of placental CD46 when the genotypes were GA or AA. However, complement deposition on placentas measured by amounts of C3b, C4b and MAC showed significantly higher levels in PE compared to normotensive pregnancies. These data suggested that SNPs in fetal complement regulators alone are not sufficient to induce complement activation on placentas.

Among factor H SNPs, rs800292 is a missense mutation (I62V) which was shown to be associated with polypoidal choroidal vasculopathy^24^. The C3b binding affinities of I62 and V62 factor H proteins were similar^25^. However, the cofactor activity of I62 factor H in factor-I mediated cleavage of C3b was slightly higher than that of V62 factor H^26^. Nevertheless, subtle decrease in cofactor function of I62V mutation may not be sufficient by itself to increase complement activation substantially but it may have an additive effect in the presence of other complement gene mutations. In our cohort, allele frequencies of rs800292 were not significantly different between PE and normotensive groups. However, in combination with fetal CD46 SNPs, rs800292 hetero/homozygous genotypes showed significantly higher frequency in PE pregnancies suggesting that it had an additive effect on complement activation. Other two SNPs in factor H rs1061147 and rs2274700 were synonymous. These two SNPs have been shown to be associated with renal and pulmonary dense deposit disease^27^. They were also shown to be in LD with rs800292 and form protective or risk haplotypes in causing AMD based on other SNPs involved^28^. In our study, allele frequencies of these two SNPs were not different between controls and cases. However, hetero/homozygous genotypes of these two SNPs formed risk complotypes in combination with fetal CD46 SNPs. Since both rs1061147 and 2274700 are synonymous they may not be the causal SNPs and may not increase complement activation independently. The association of complotypes consisting of these two SNPs with PE in our analysis could be due to their LD with missense mutation rs800292.

Of the four SNPs identified in C3 gene, two were part of risk complotypes with their hetero/homozygous genotypes in combination with homozygous genotype of CD46 SNPs showing significantly higher frequency in PE pregnancies. Among the two C3 SNPS of the risk complotypes, rs2230205 was intronic whereas rs344555 was synonymous. The rs344555 was associated with serum C3 levels in systemic lupus erythematosus patients of white European population^29^ with a positive correlation between rare allele and serum C3 levels in that population. However, in a study involving Han Chines population rs344555 was not associated but a different SNP which is not part of the rs344555 haplotype block was associated with serum C3 levels. It was speculated that these two SNPs may represent different tagging SNPs for an unknown causal SNP^30^. Interestingly, in a recent study the rs223005 was shown to be  associated with severe PE^18^.

The A allele of rs800292 (maternal factor H) showed a dosage effect on maternal AP50 values irrespective of PE status. However, higher maternal AP50 was observed in both PE and normotensive groups when the genotypes of rs800292 were GA or AA. Therefore, increased AP potential alone may not be a causal factor in increased complement activation in PE. On the other hand, higher A allele frequency of rs800282 (0.53 v/s 0.30, statistically not significant) may contribute to higher maternal AP50 in PE. Overall, these data suggested that when present alone, the fetal CD46 or maternal CFH variants were not sufficient to increase placental complement deposition or maternal AP50 values respectively, since these variants were present in both PE and control subjects. However, combinations of these variants in the form of complotypes were associated with increased complement activity in PE. The limitation of our study was the small sample sizes of PE and normotensive groups and therefore, validation in an independent larger cohort is needed. Further, we could not analyze complotypes of higher orders (3 or more SNPs) due to inadequate number of individuals representing each potential complotype to obtain statistically meaningful outcome. Rare variants of complement genes were not analyzed due to small sample size. We could not test if complotype associations remain significant after adjusting for known covariates in PE such as maternal high BMI, advanced maternal age (≥35 years) and prior PE. If these findings are relevant for women from different ethnicities remains to be tested. However, this study is also important since there are only a few studies on black and Hispanic women.

In summary, the data obtained in this study collectively demonstrate the concept of pregnancy specific complotypes and their potential association with PE. Further research is needed to understand if common and rare variants of complement genes predict different PE phenotypes such as late-onset mild and early-onset severe respectively. In addition, SNPs in other complement genes such as maternal CFI, C5, C6-9 and fetal CD55 and CD59 may constitute independent or extended risk complotypes for PE. Thus, our findings in this study form the basis for the design and testing in a larger cohort to demonstrate complotype predisposition in women to develop PE with important clinical implications for the early detection, management and treatment of PE. 

## Methods

### Study population

We performed a retrospective study to identify and compare frequencies of SNPs in 16 PE and 24 normotensive pregnancies. Study subjects were selected from perinatal database and biospecimen repository (PeriBank), Baylor College of Medicine^31^. The tissue bank collects samples from consenting women who present to the labor and delivery unit of Ben Taub hospital and the Texas Children’s hospital Pavilion for Women. All the samples collected between 2012 and 2015 for which matched placenta, maternal blood and fetal cord blood were available and met our inclusion and exclusion criteria were included in the analysis. Our exclusion criteria for specimen were known antenatal infections, autoimmune disorders, abnormal placentation, chronic hypertension or renal disease, atherosclerotic or vascular disorders, or fetal structural or chromosomal disorders. Our inclusion criteria were singleton pregnancies from non-morbid obese and non-obese (pre-pregnancy BMI < 40) gravidae, ≤35 years old, delivered by either C-section or vaginal and with or without IUGR. In PE group 7 specimens were from early-onset severe cases and 9 specimens were from mild PE.

### C3, factor H and CD46 sequencing

The exons including flanking intronic regions were sequenced using standard Sanger sequencing. We used primers for C3 as previously reported^18^. The primers used for factor H and CD46 are listed in Supplemental Tables 1 and 2. PCR was performed using GoTaq Green Master Mix (PROMEGA, USA) as per the instruction manual. 25 µl reactions were set up with 1 µl DNA template (<250 ng) and 10 µM Primers. The reaction mixture was incubated at 94 °C for 3 min and cycled according to the following parameters: 94 °C for 30 s and 60 °C for 30 s for a total of 40 cycles and a final extension at 72 °C for 10 minutes. The PCR products were run in 1% Agarose gel for verification and purified using spin QIAquick column method (QIAGEN, USA). An ABI 3130xl was used for sequencing and the variant analysis was carried out by Sequencher 5.4.6 software (Gene Codes, USA)

### Complement deposition on placentas

C3b, C4b and MAC levels on placentas were determined by enzyme linked immune assays using specific kits as per the instructions provided by the manufacturers.

### Western blot

Placental tissue lysates containing 20 μg protein were electrophoresed on 5–20% (w/v) polyacrylamide gradient gels (NOVEX, Life Technologies, USA) and transferred on to Immune-Blot PVDF membranes (Bio-Rad, USA). Expression levels of CD46 were probed using rabbit monoclonal antiCD46 antibody (ABCAM, USA). A secondary antibody goat anti-rabbit IgG-HRP (SouthernBiotech, USA) and pierce ECL Western blotting substrate kit (Thermo Fisher Scientific, USA) were used to develop the membranes. The Western bands were visualized using Odyssey IR imaging system (LI-COR Biosciences, USA).

### AP50 and CH50

One AP50 unit is defined as the volume of serum required to destroy 50% of rabbit erythrocytes. One CH50 unit is defined as the volume of serum required to destroy 50% of antibody sensitized sheep erythrocytes. The reagents for AP50 and CH50 assays were purchased from Complement Technologies, Inc (USA) and assays were performed as per the instructions from the manufacturers. Briefly, for AP50 assay maternal sera were diluted 1:2 and then dispensed to 9 wells in a microtiter plate with increasing volume from 2 to 30 µl. To each well 2.5 µl of rabbit erythrocytes (5 × 10^8^ cells/mL in GVB° buffer) and 5 µl 0.1 M MgEGTA were added and the volume was made up to 100 µl by GVB° buffer. Two background wells, one with 20 µl serum and other without were also set up. One well was set up for maximum lysis with 20 µl and 55 µl of maximum lysis buffer (GVB° + 0.2% NP40). Contents were mixed on ice and microplate was transferred to 37 °C incubator. Contents were mixed approximately every 5 minutes. After 30 minutes microplate was centrifuged for 3 minutes at 1000 × g to pellet cells. The supernatant was read at 412 nm. From each reading background was subtracted and then divide by maximum lysis minus background and multiplied by 100 to get % of maximum lysis. % of maximum lysis was plotted against volume of ½ serum and the volume that yielded 50% lysis was determined. This volume contains 1 AP50 unit of complement activity. The AP50 units per mL of undiluted serum was then calculated.

For CH50 assay maternal sera were dilute 1:100 and then dispensed then dispensed to 6 wells in a microtiter plate with increasing volume from 30 to 100 µl. To each well 20 µl of sheep erythrocytes coated with optimum levels of rabbit anti-sheep erythrocyte IgM antibodies were added and the volume was made up to 150 µl by GVB^++^ buffer. Two negative controls without serum were set up. Two 100% controls with water but without serum and GVB^++^ were set up. Contents were mixed approximately every 10 minutes. After 60 minutes microplate was centrifuged for 3 minutes at 1000 × g to pellet cells. The supernatant was read at 541 nm. Absorbance (OD) of two negative and two 100% controls were averaged. Average negative control values were subtracted from all other readings including 100% controls to obtain background corrected reading for each well. The proportion of cells lysed (y) was calculate as follows, y = corrected OD of each assay well/corrected OD of averaged 100%. log y/1-y was then calculated for each assay well and plotted against log of volume of dilute serum. Volume of diluted serum at which y/1-y equals 1.0 yielded 50% hemolysis and contained 1 CH50 unit of complement activity. The CH50 units per mL of undiluted serum was then calculated. For both AP50 and CH50 each maternal serum was measured in triplicates and average of three was presented.

### Statistics

The association of minor allele frequencies and complotypes with PE were analyzed using Fisher’s exact test. For all other values the data was presented as mean ± SEM with triplicate measurements. Differences between two groups were analyzed by student t test followed by Mann-Whitney whereas ordinary ANOVA was used for more than two groups. A *p* value of less than 0.05 was considered to be significant.

### Study approval

This study was approved by the Baylor College of Medicine institutional review board and conducted according to Declaration of Helsinki principles. Written informed consent was obtained from all participants prior to inclusion in the tissue bank.

## Supplementary information

Supplementary information.
