# Supplementary material for: Common variants of fetal and maternal complement genes in preeclampsia: pregnancy specific complotype
Source: Sci Rep. 2020 Mar 16;10:4811. doi: 10.1038/s41598-020-60539-9 (PMC7076030; doi:10.1038/s41598-020-60539-9)
Supplement: Supplementary file 1 — Supplementary information. [file 41598_2020_60539_MOESM1_ESM.pdf]

Common variants of fetal and maternal complement genes in preeclampsia: pregnancy specific complotype.

Exon	Forward	Reverse
1	TTCTGTTAGGTTCCGCCAGG	AGAACCCTGTCCCCAAACAG
2	CACTGCTATGAGCACTCAGGT	AGAAAATCATCATCACCGTAGTGGA
3	ATTCCCACCCATTCAAAAGAGC	CATCCAAGAGTTGTTTGGCTAGA
4	ACCACCCCCTCAAACTACTG	ACTCGGTGCTAGTTAAGAAATCC
5	TCACAAACAGCTTGTAGAAACC	CAGGAGGAGGAAGCACATACA
6	TTCCTTGTCTCTGTTCACACTGG	GCAACAACAATAACAAACCAAGACA
7 - 8	TCCAGGGTTCTTAGCACGTTA	ACAAATGTCCTCCCTCCTTTCA
9-10	ACAGAAAATGTGAGTGGGGAGG	GATCCTATGTTTGGGCACCTCA
11 - 12	TGCTATCTGGAGATCCATGTGTT	TGAAGCTGCACAAAAGCATGT
13	ACCGGTACCATTTCTCCCCA	TGCCAATATCTCTTTGCTCAGG
14a	AGGCATCACATACTTGAACAGT	AGCCAAGTTTGTGCATATTCC
14b	TACTACTTTGGCTCTTGTGCA	GAAATGCAGTGGCAGTCCCT
14c	CAGCCCTCTACTGAGTCCCT	GTGGAGAAGCCTGGGGATA

Supplementary Table 1. Primers used in the sequencing of CD46 gene

Exon	Forward	Reverse
1	TGTCTGGGTGCTGATTGTGAA	TCCTGTGAAAAGCATCATTAGCA
2	TCCTCTTTGCTCCCACAACT	CTGGCAATAGTGATATAATTCAGGC
3	ACTTGTTCCCCCACTCCTACA	TAAACCTCTTTTCGTATGGACTACA
4	TGGAGGCAAGTGCTGAAAGT	AAAGACTAGATTCCCACTCTACATT
5	CCACTCCCATAGAAAAGAATCAGG	ACTTCTTTGCACCAGTCTCTTCC
6	AGCGGTCAAGTCAAAACAGA	CCTCACTCTGATCCAGACATGG
7	GGATTAAGAGCCCAGGAGGT	ATGCTTCCAACAGCCTTACTT
8	GAGCTAAGCGGTAAAATTGGCA	TGTGCTCTCCTTTCTTCGATCTT
9	GTTGTTCAAGCAAAGTGACCA	CCATTGGTAAAACAAGGTGACAT
10	ATGTCTTTGGCAACTCTGAGC	ATCAGCCCCCACAAAAAGAC
11	ACGACAACAAATTCTCACCAGT	AGGTAGGTGGCCCATAGGAA
12	ATGCCCCTCTGTATGACCCA	GCAATGGGAGCCCAAACAAA
13-14	TGATCAAATGCTTGCCTCAGTT	TGAAGACTGGAAATGTTGAGGC
15	TCAGCGACAGAATACAGGGC	TCATTAGTTTCTAGTTTACCTGCCT
16	GCAATGCATTAAACTGGGTGC	TTGTTTACACGAAGCACAAGAG
17	TGGTGGAGGAATATATCTTTGCGA	CCCCTCACTTTGATAACAAGAGATT
18	GACAGACAGACACCAGAAGGC	AATTTCCCACAGCAGTCCAGAAT
19	TCTCAATTGCTACGGCTACCA	CGACTACTTTGTCCTGCCGT
20	TCAGTGTTCTAGCGAAGGATGA	TTTAACCCTGCTATACTCCCCCA
21	ATACAGTGCTGTGTTTGCGT	GAGATTTTTCCAGCCACGTGAA
22	ACGCAGGGATCCTAAAATGACA	AGTGAGCCTCAGAAGCTGTG

Supplementary Table 2. Primers used in the sequencing of factor H gene

Figure 1. Full length Westerns of placental CD46 expression

Western 1

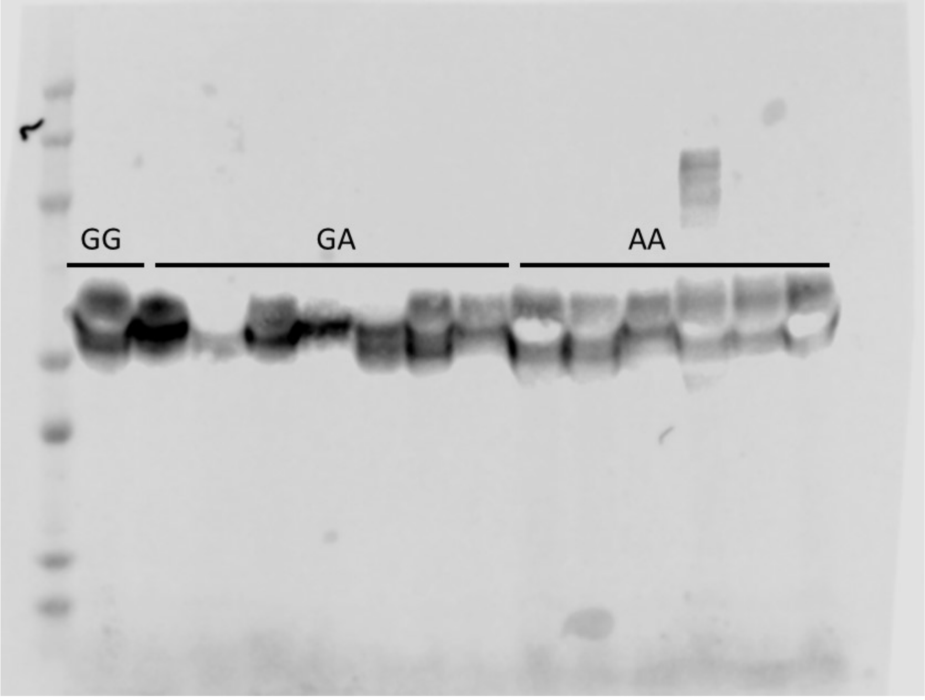

CD46

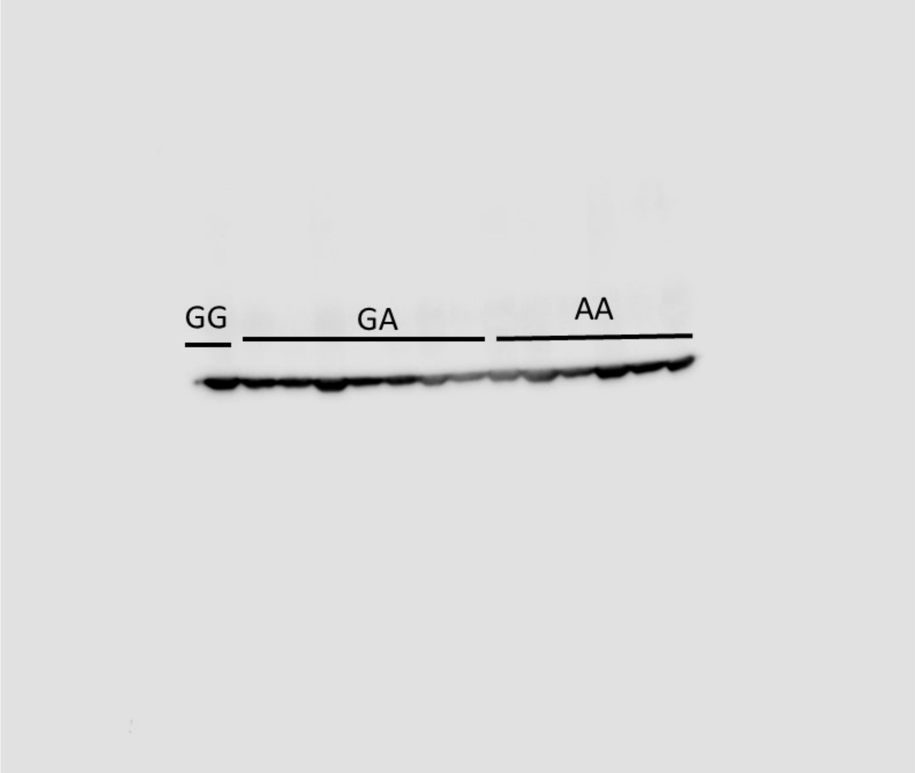

β-actin

Western 2

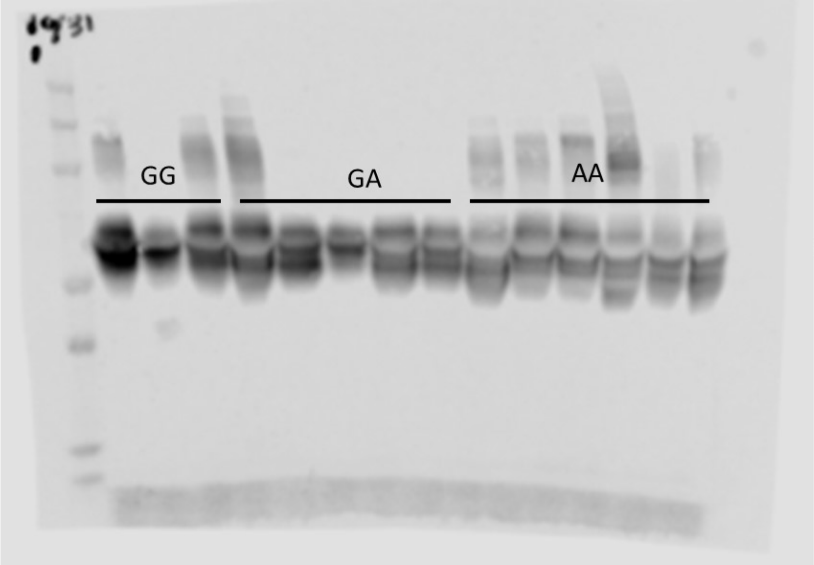

CD46

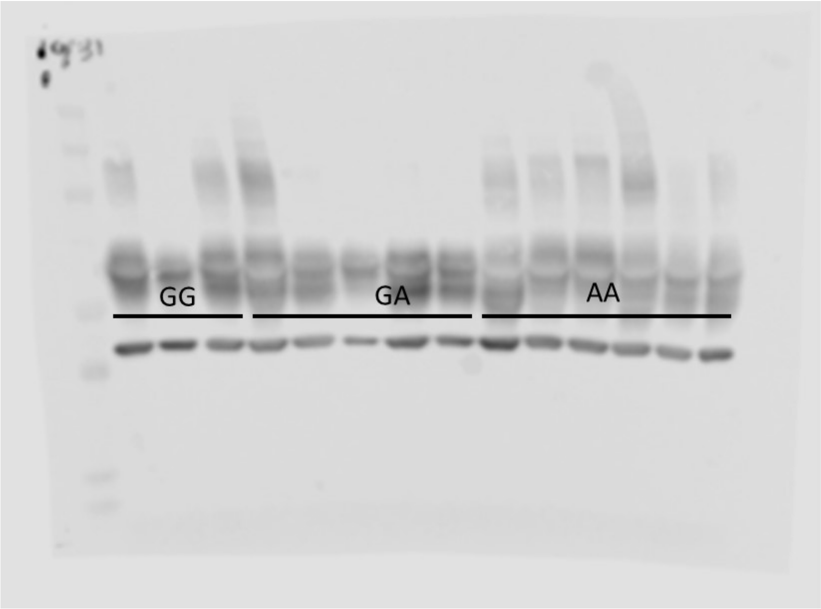

β-actin

Western 3

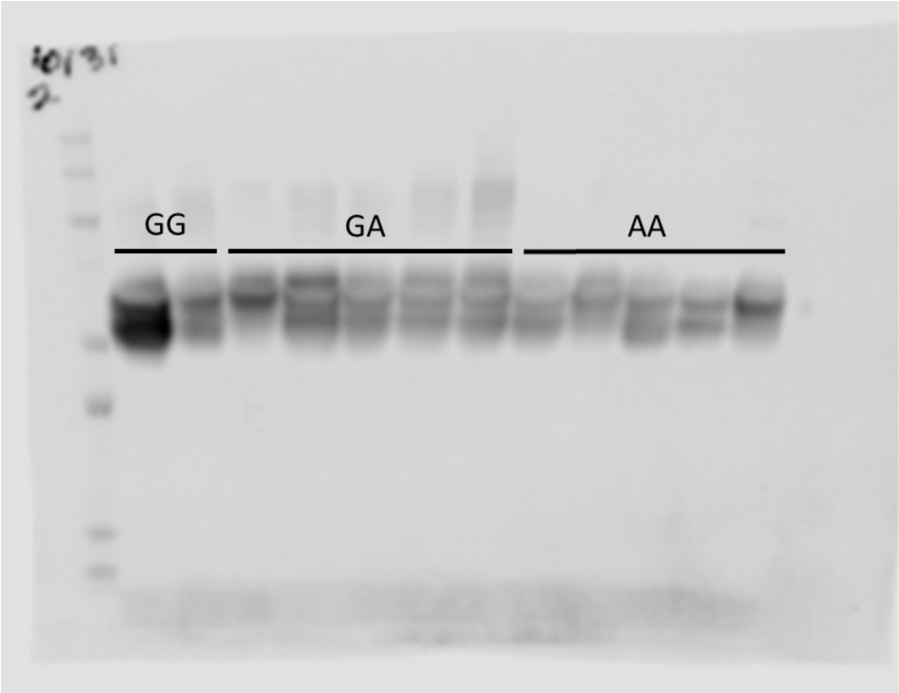

CD46

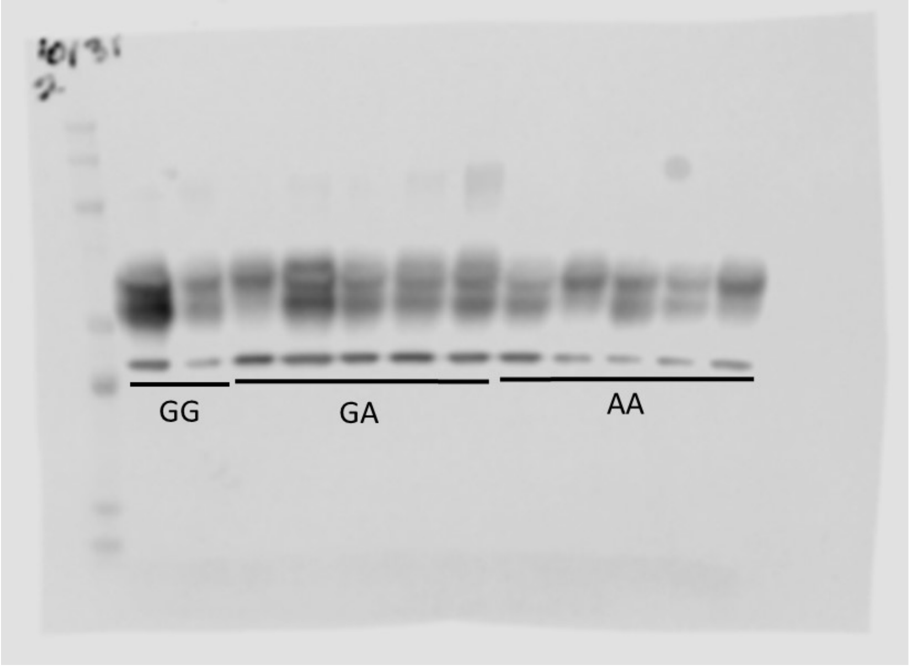

β-actin
